# Multiple Desirable Methods in Outlier Detection of Univariate Data With R Source Codes

**DOI:** 10.3389/fpsyg.2021.819854

**Published:** 2022-01-17

**Authors:** Yuho Shimizu

**Affiliations:** Graduate School of Humanities and Sociology, The University of Tokyo, Tokyo, Japan

**Keywords:** outlier, square root transformation, median absolute deviation, Grubbs' test, Ueda's method, univariate data

## Introduction

The existence of outliers has been a methodological obstacle in various literature (Grubbs, 1969; Tian et al., 2018; Erdogan et al., 2019). There are many cases when we should deal with outliers of univariate data. If inappropriate methods are used, it can lead to biased and wrong conclusions (Aguinis et al., 2013; Fife, 2020). Hence, how to detect outliers is one of the hottest topics among researchers in many fields (Tian et al., 2018; Dutta and Banerjee, 2019; Saneja and Rani, 2019), including psychology (Gladwell, 2008; Blouvshtein and Cohen-Or, 2018; Leys et al., 2019).

Although outlier detection methods should be considered enough in psychology, many researchers have used inappropriate methods without any theoretical basis (Simmons et al., 2011; Leys et al., 2013; Obikee and Okoli, 2021). Leys et al. (2013) investigated outlier detection methods in 127 articles published in Journal of Personality and Social Psychology (JPSP) and Psychological Science (PSS) from 2010 to 2012. As a result, 56 papers (about half of the 127 papers) used the outlier detection methods with the mean and standard deviation (Leys et al., 2013). I call the method “the conventional method” in this article. In this method, outliers are the values which do not fall within the mean ± *x* times standard deviation (*x* = 2 or 2.5 are common; Leys et al., 2013; Yang et al., 2019). Because of its simplicity, this method has been used in a great many psychological studies (Simmons et al., 2011; Leys et al., 2013).

However, the conventional method has the three major theoretical problems (Chiang et al., 2003; Simmons et al., 2011). First, a normal distribution is assumed including outliers (Miller, 1991; Yang et al., 2019). Second, the mean and standard deviation are highly skewed by outliers and it leads to increasing the likelihood of Type I and Type II errors (Cousineau and Chartier, 2010; Leys et al., 2013). Third, it is difficult to detect outliers in data with a small sample size (Cousineau and Chartier, 2010).

As shown above, the conventional method has several theoretical problems, but it has been used in many studies without sufficient consideration (Simmons et al., 2011; Leys et al., 2013; Obikee and Okoli, 2021). There are two possible reasons for this situation. First, there are not many known more appropriate methods other than the conventional method. Second, how to perform those desirable methods is not fully understood by researchers. Each researcher should choose the method that is appropriate for data.

The purpose of this opinion paper is reviewing more desirable methods for detecting outliers of univariate data (specifically, square root transformation, median absolute deviation, Grubbs' test, and Ueda's method), and presenting source code and sample data that allow us to conduct each detection method. These detection methods have desirable advantages over the conventional method and they are relatively easy to implement. In addition, the results of applying each outlier detection method to a real data set are shown. Presented methods in this article can be conducted using R (R Core Team, 2021), a free statistical software. By summarizing various outlier detection methods and providing analysis source codes, useful knowledge in psychological research can be provided.

## Outlier Detection Methods

### Square Root Transformation

The method of square root transformation can be used for the biased data with which normal distribution cannot be assumed, but it cannot be used for data that are too asymmetric (Cousineau and Chartier, 2010). When dealing with extreme asymmetric data, please refer to Carling (2000). First, the data *x* is transformed according to the following equation (1).


(1) $$\begin{array}{l} y=\;\sqrt{\frac{x-Xmin}{Xmax-Xmin}} \end{array}$$

In equation (1), *x* is each data, *Xmin* is the minimum value of the data, and *Xmax* is the maximum value of the data. The data *y* is a number falling between 0 and 1. In the square root transformation, the *z*-score is calculated by equation (2), for the data *y*.


(2) $$\begin{array}{l} z=\;\frac{y-Ym}{Sy} \end{array}$$

In equation (2), *Ym* is the mean of *y* and *Sy* is the standard deviation of *y*. A robust *z*-score transformation has higher power in detecting outliers. Then, the outlier is determined by Bonferroni correction (Armstrong, 2014). The Bonferroni correction is performed to avoid Type II errors that may occur in response to a larger standard deviation (Cousineau and Chartier, 2010). The *z*-values before and after Bonferroni correction for a representative sample size *N* were shown in the Open Science Framework repository (OSF; https://osf.io/szt5n/?view_only=5cd1c734b392442d9633d3b7414c0914).

### Median Absolute Deviation

The method of using median absolute deviation (*MAD*) was proposed by Hampel (1974) and can be used for the biased data with which normal distribution cannot be assumed, but the method is not yet common in psychological research (Leys et al., 2013). The statistic *MAD* uses the median, which has a very desirable characteristic that it is stable against the influence of outliers (Leys et al., 2013; Yang et al., 2019). *MAD* is obtained by the following equations (3) and (4).


(3) $$\begin{array}{l} MAD=b\;Med\left(\left|x-Med\left(x\right)\right|\right) \end{array}$$

(4) $$\begin{array}{l} b=\frac{1}{Q\left(0.75\right)} \end{array}$$

*Med(x)* denotes the median value in data *x*. *Q*(0.75) refers to the 75th percentile (third quartile) of *z*-scores. When a normal distribution can be assumed, *b* = 1/*Q*(0.75) = 1.4826 is often used (Huber, 1981; Leys et al., 2013; Kannan et al., 2015). Then, the median ± *k* times of *MAD* is considered to be the border of outliers. For example, Miller (1991) recommends using 2, 2.5, or 3 as the value *k*, depending on the purpose of outlier detection, while Leys et al. (2013) recommend a criterion of 2.5 as the value *k*. By adjusting the coefficient *b*, it is possible to use this method when normal distribution is not assumed (e.g., those with high kurtosis), but robust detection cannot be achieved for extremely asymmetric data (Rousseeuw and Croux, 1993; Yang et al., 2019). The method of using *MAD* is shown that it is reasonable with Carling's modification of the boxplot rule, and please also see Wilcox (2006) and Ng and Wilcox (2010). Source codes of the method of *MAD* uses *stats* package of R (R Core Team, 2021).

### Grubbs' Test

When the Grubbs' test (Grubbs, 1950) is conducted, normal distribution should be assumed. On the contrary, it has an advantage that removed outliers have no effect on the next outlier detection. Specifically, the statistic *T* is calculated for the maximum and minimum value of the data, respectively, and is tested against the significance level α set for the sample size *N* (Ahmed et al., 2020). The statistic *T* is obtained by the following equation (5) or (6).


(5) $$\begin{array}{l} T=\frac{Xmax-Xm}{Sx} \end{array}$$

(6) $$\begin{array}{l} T=-\frac{Xmin-Xm}{Sx} \end{array}$$

*Xmax* is the maximum value, *Xm* is the mean value, *Xmin* is the minimum value, and *Sx* is the standard deviation of the data. A test is performed on the maximum and minimum value, respectively. If it is judged to be significant, the value is removed from the analysis, as an outlier. Then, the test is repeated. However, the repetition of such tests leads to the problem of multiple comparisons where the probability of Type I error exceeds the significance level α (Jain, 2010). To deal with the problem, the Bonferroni correction should be used. Source codes of Grubbs' test use *outliers* package (Lukasz, 2011).

### Ueda's Method

Ueda's method (Ueda, 1996/2009) can be used for the biased data with which normal distribution cannot be assumed (Marmolejo-Ramos et al., 2015b). This method uses Akaike's Information Criterion (AIC), and the statistic *Ut* is calculated by the following equation (7).


(7) $$\begin{array}{l} Ut=\;\frac{1}{2}\;AIC\;≅n\;\log\hat{\sigma }+\;\sqrt{2}\;s\;\frac{\log n!}{n} \end{array}$$

*n* is the number of data considered not to be outliers, *s* is the number of data considered to be outliers, and the total sample size *N* is represented by *n* + *s*. $\hat{\sigma }$ is the standard deviation of the data considered not to be outliers. In this method, the original data *x* is first converted into *z*-scores using the equation (2), and then equation (7) is applied to the *z*-scores to obtain *Ut*. We select the data that seem to be outliers and calculate *Ut* for each case. When the statistic *Ut* is minimized, *s* is the number of outliers and the omitted data is detected as outliers [see Ueda (1996/2009) and Marmolejo-Ramos et al. (2015b) for more detailed methods]. Ueda's method is relatively simple (Marmolejo-Ramos et al., 2015b) and have an advantage that it can be used regardless of the sample size *N* (Ueda, 1996/2009).

## R Source Codes and Sample Data

In this article, applicable R source codes and sample data are provided. These can be downloaded from OSF. For Ueda's method, please also refer to the useful R code by Marmolejo-Ramos et al. (2015b). R is a free software and my source codes can be easily applied to univariate data in several fields, which might be a practical contribution for many researchers.

Sample data was obtained in April 2020. Participants were university students at the author's affiliation and participated as volunteers. They were asked a single question item, “How many times do you think you have taken a train in your life?” Participants lived in an urban area with many railroads. Therefore, this item was thought to be suited for the objective to detect outliers that were too small or too large. This data and source codes allow us to practice outlier detection methods described above, and the summary of the results was posted on OSF. In addition, the results of applying each outlier detection method to a real data set (*Fisher's Iris* data set in R) were posted on OSF. It was shown that the values considered as outliers differed greatly depending on each method.

## Discussion

Four effective methods for detecting outliers of univariate data were reviewed in this article. Furthermore, R source codes that can be used for each method were provided along with sample data. In this article, outlier detection methods for univariate data were provided, and for multivariate data, please refer to Hadi (1992) and Rocke and Woodruff (1996). It is said that outlier detection methods for univariate data can often be applied in the case of multivariate data (Pan et al., 2000; Bauder and Khoshgoftaar, 2017), and thus, this article has a high potential for use. Although this research has not been verified by the simulation approach, Marmolejo-Ramos et al. (2015a) conducted and verified several outlier detection methods by the simulation. The comparison of the outlier detection methods by such advanced technique is very meaningful and should be referred to by many researchers. As noted above, despite the several theoretical problems in the conventional method, it has been used in many psychological studies without enough consideration (Simmons et al., 2011; Leys et al., 2013; Obikee and Okoli, 2021). Scientists should choose an appropriate outlier detection method along with their data.

Another major problem is that a certain number of studies do not report which outlier detection method was used. Leys et al. (2013) reviewed outlier detection methods of 127 papers and raised an alarm about the existence of 37 papers which did not describe outlier detection methods. In the future, it should be clearly stated whether outliers have been considered and the details of the detection method (Leys et al., 2013). Furthermore, since each method has its own advantages and disadvantages (Yang et al., 2019; Ahmed et al., 2020; Satari and Khalif, 2020), we should have a clear understanding of the characteristics of the data before choosing which detection method to use. It is necessary to become familiar with a wide range of detection methods, including those not covered in this study, and use them according to the data.

All four methods reviewed in this article can be easily replicated by R source codes on OSF. In a similar effort to this article, Thompson (2006) published a method for detecting outliers in univariate data using the statistical software SPSS, and the source code is freely downloaded. If user-friendly tools become widely available, the number of cases where “*Conventional methods are used for now*” will decrease. Each academic researcher needs to strive to use appropriate methods in outlier detection.

## Author Contributions

YS: article development, composition, draft review, and creative oversight.

## Conflict of Interest

The author declares that the research was conducted in the absence of any commercial or financial relationships that could be construed as a potential conflict of interest.

## Publisher's Note

All claims expressed in this article are solely those of the authors and do not necessarily represent those of their affiliated organizations, or those of the publisher, the editors and the reviewers. Any product that may be evaluated in this article, or claim that may be made by its manufacturer, is not guaranteed or endorsed by the publisher.

## References

[B1] Aguinis H. Gottfredson R. K. Joo H. (2013). Best-practice recommendations for defining, identifying, and handling outliers. Organ. Res. Methods 16, 270–301. 10.1177/1094428112470848

[B2] Ahmed A. Khan M. S. Gul N. Uddin I. Kim S. M. Kim J. (2020). A comparative analysis of different outlier detection techniques in cognitive radio networks with malicious users. Wirel. Commun. Mob. Comput. 2020:8832191. 10.1155/2020/8832191

[B3] Armstrong R. A. (2014). When to use the Bonferroni correction. Ophthalmic Physiol. Opt. 34, 502–508. 10.1111/opo.12131 24697967

[B4] Bauder R. A. Khoshgoftaar T. M. (2017). Multivariate outlier detection in medicare claims payments applying probabilistic programming methods. Health Ser. Outcomes Res. Methodol. 17, 256–289. 10.1007/s10742-017-0172-1

[B5] Blouvshtein L. Cohen-Or D. (2018). Outlier detection for robust multi-dimensional scaling. IEEE Trans. Pattern Anal. Mach. Intell. 41, 2273–2279. 10.1109/TPAMI.2018.2851513 29994700

[B6] Carling K. (2000). Resistant outlier rules and the non-Gaussian case. Comput. Stat. Data Anal. 33, 249–258. 10.1016/S0167-9473(99)00057-2

[B7] Chiang L. H. Pell R. J. Seasholtz M. B. (2003). Exploring process data with the use of robust outlier detection algorithms. J. Process Control 13, 437–449. 10.1016/S0959-1524(02)00068-9

[B8] Cousineau D. Chartier S. (2010). Outliers detection and treatment: a review. Int. J. Psychol. Res. 3, 59–68. 10.21500/20112084.844

[B9] Dutta J. K. Banerjee B. (2019). Improved outlier detection using sparse coding-based methods. Pattern Recognit. Lett. 122, 99–105. 10.1016/j.patrec.2019.02.022

[B10] Erdogan B. Hekimoglu S. Durdag U. M. Ocalan T. (2019). Empirical estimation of the power of test in outlier detection problem. Stud. Geophys. Geod. 63, 55–70. 10.1007/s11200-018-1144-9

[B11] Fife D. (2020). The eight steps of data analysis: a graphical framework to promote sound statistical analysis. Perspect. Psychol. Sci. 15, 1054–1075. 10.1177/1745691620917333 32502366

[B12] Gladwell M. (2008). Outliers: The Story of Success. New York, NY: Little, Brown.

[B13] Grubbs F. E. (1950). Sample criteria for testing outlying observations. Ann. Math. Stat. 21, 27–58. 10.1214/aoms/1177729885

[B14] Grubbs F. E. (1969). Procedures for detecting outlying observations in samples. Technometrics 11, 1–21. 10.1080/00401706.1969.10490657

[B15] Hadi A. S. (1992). Identifying multiple outliers in multivariate data. J. R. Stat. Soc. Series B 54, 761–771. 10.1111/j.2517-6161.1992.tb01449.x

[B16] Hampel F. R. (1974). The influence curve and its role in robust estimation. J. Am. Stat. Assoc. 69, 383–393. 10.1080/01621459.1974.10482962

[B17] Huber P. J. (1981). Robust Statistics. New York, NY: John Wiley. 10.1002/0471725250

[B18] Jain R. B. (2010). A recursive version of Grubbs' test for detecting multiple outliers in environmental and chemical data. Clin. Biochem. 43, 1030–1033. 10.1016/j.clinbiochem.2010.04.071 20471967

[B19] Kannan K. S. Manoj K. Arumugam S. (2015). Labeling methods for identifying outliers. Int. J. Stat. Sys. 10, 231–238.

[B20] Leys C. Delacre M. Mora Y. L. Lakens D. Ley C. (2019). How to classify, detect, and manage univariate and multivariate outliers, with emphasis on pre-registration. Int. Rev. Soc. Psychol. 32, 1–10. 10.5334/irsp.289

[B21] Leys C. Ley C. Klein O. Bernard P. Licata L. (2013). Detecting outliers: do not use standard deviation around the mean, use absolute deviation around the median. J. Exp. Soc. Psychol. 49, 764–766. 10.1016/j.jesp.2013.03.013

[B22] Lukasz K. (2011). Outliers: Tests for Outliers.

[B23] Marmolejo-Ramos F. Cousineau D. Benites L. Maehara R. (2015a). On the efficacy of procedures to normalize Ex-Gaussian distributions. Front. Psychol. 5:1548. 10.3389/fpsyg.2014.01548 25709588 PMC4285694

[B24] Marmolejo-Ramos F. Vélez J. I. Romão X. (2015b). Automatic outlier detection via the Ueda method. J. Stat. Distrib. Appl. 2, 1–14. 10.1186/s40488-015-0031-y

[B25] Miller J. (1991). Reaction time analysis with outlier exclusion: bias varies with sample size. Q. J. Exp. Psychol. 43, 907–912. 10.1080/14640749108400962 1775668

[B26] Ng M. Wilcox R. R. (2010). The small-sample efficiency of some recently proposed multivariate measures of location. J. Mod. Appl. Stat. Methods 9, 28–42. 10.22237/jmasm/1272686640

[B27] Obikee A. C. Okoli C. N. (2021). Evaluation of some outlier detection methods based on real life data application. Int. J. Innov. Sci. Eng. Technol. 4, 81–90.

[B28] Pan J. X. Fung W. K. Fang K. T. (2000). Multiple outlier detection in multivariate data using projection pursuit techniques. J. Stat. Plan. Inference 83, 153–167. 10.1016/S.0378-3758(99)00091-9

[B29] R Core Team (2021). R: A Language and Environment for Statistical Computing. Vienna: R Foundation for Statistical Computing.

[B30] Rocke D. Woodruff D. (1996). Identification of outliers in multivariate data. J. Am. Stat. Assoc. 91, 1047–1061. 10.1080/01621459.1996.10476975

[B31] Rousseeuw P. Croux C. (1993). Alternatives to the median absolute deviation. J. Am. Stat. Assoc. 88, 1273–1283. 10.1080/01621459.1993.10476408

[B32] Saneja B. Rani R. (2019). A scalable correlation-based approach for outlier detection in wireless body sensor networks. Int. J. Commun. Syst. 32:e3918. 10.1002/dac.391825855820

[B33] Satari S. Z. Khalif K. M. N. K. (2020). Review on outliers identification methods for univariate circular biological data. Adv. Sci. Technol. Eng. Syst. 5, 95–103. 10.25046/aj050212

[B34] Simmons J. P. Nelson L. D. Simonsohn U. (2011). False-positive psychology: undisclosed flexibility in data collection and analysis allows presenting anything as significant. Psychol. Sci. 22, 1359–1366. 10.1177/0956797611417632 22006061

[B35] Thompson G. (2006). An SPSS implementation of the nonrecursive outlier deletion procedure with shifting z score criterion (Van Selst & Jolicoeur, 1994). Behav. Res. Methods 38, 344–352. 10.3758/BRM.38.2.344 16956111

[B36] Tian Y. Yin Z. Huang M. (2018). Missing data probability estimation-based Bayesian outlier detection for plant-wide processes with multisampling rates. Symmetry 10:475. 10.3390/sym10100475

[B37] Ueda T. (1996/2009). A simple method for the detection of outliers. Electronic J. Appl. Stat. Anal. 2, 67–76.

[B38] Wilcox R. R. (2006). Comparing robust generalized variances and comments on efficiency. Stat. Methodology 3, 211–223. 10.1016/j.stamet.2005.09.005

[B39] Yang J. Rahardja S. Fränti P. (2019). “Outlier detection: how to threshold outlier scores?” in Proceedings of the International Conference on Artificial Intelligence, Information Processing and Cloud Computing, 1–6. 10.1145/3371425.337142725911276

